# Functional characterization of a small heat shock protein from *Mycobacterium leprae*

**DOI:** 10.1186/1471-2180-8-208

**Published:** 2008-11-28

**Authors:** Nirmala Lini, Elengikal Abdul Azeez Rehna, Sugathan Shiburaj, Jayapal Jeya Maheshwari, Nallakandy Panagadan Shankernarayan, Kuppamuthu Dharmalingam

**Affiliations:** 1 Department of Genetic Engineering, School of Biotechnology, Madurai Kamaraj University, Madurai-625 021, Tamil Nadu, India; 2 Applied Microbiology and Microbial Technology Lab, Tropical Botanical Garden Research Institute, Palode, Thiruvanathapuram-695562, Kerala, India; 3 Voluntary Health Services, Leprosy Project, Shaktinagar, Periyar District-638315, Tamil Nadu, India

## Abstract

**Background:**

Small heat shock proteins are ubiquitous family of stress proteins, having a role in virulence and survival of the pathogen. *M. leprae*, the causative agent of leprosy is an uncultivable organism in defined media, hence the biology and function of proteins were examined by cloning *M. leprae *genes in heterologous hosts. The study on sHsp18 was carried out as the knowledge about the functions of this major immunodominant antigen of *M. leprae *is scanty.

**Results:**

The gene encoding *Mycobacterium leprae *small heat shock protein (sHsp18) was amplified from biopsy material of leprosy patients, and cloned and expressed in *E. coli*. The localization and *in vitro *characterization of the protein are detailed in this report. Data show that major portion of the protein is localized in the outer membrane of *E. coli*. The purified sHsp18 functions as an efficient chaperone as shown by their ability to prevent thermal inactivation of restriction enzymes *Sma*I and *Nde*I. Physical interaction of the chaperone with target protein is also demonstrated. Size exclusion chromatography of purified protein shows that the protein can form multimeric complexes under *in vitro *conditions as is demonstrated for several small heat shock proteins.

**Conclusion:**

The small heat shock protein sHsp18 of *M. leprae *is a chaperone and shows several properties associated with other small heat shock proteins. Membrane association and *in vitro *chaperone function of sHsp18 shows that the protein may play a role in the virulence and survival of *M. leprae *in infected host.

## Background

Small heat shock proteins are ubiquitous, and found in the cytosol of eukaryotes as well as prokaryotes. These proteins differ from other heat shock protein families because they have a conserved amino acid sequence motif called the α-crystallin domain [1]. The central α-crystallin domain, consisting of about 90 amino acids, is highly conserved. The C-terminal domain varies from 12 residues in human hsp20 to 36 residues in mouse hsp25. The N-terminal extension is highly variable among different sHsps. It has also been shown that the C-terminal extensions have a conserved LXL/V sequence in all sHsps [2,3].

The subunit molecular mass of sHsps ranges from 13 to 42 kDa. However, the functional forms of all these proteins are generally multimers. Most sHsps form multimers from the basic monomeric structures [4]. Unlike other bacterial and eukaryotic sHsps, *acr*1 gene encoded sHsp of *M. tuberculosis *forms a trimer of trimers and the functions of this protein have been studied extensively [5]. Among the sHsps studied, Acr1 and Acr2 proteins of *M. tuberculosis *have been studied in the context of pathogenesis as well. Acr1 is up regulated under stress conditions such as hypoxia and S-Nitrosoglutamine and ethanol treatment, but not under heat shock [6]. Further, *acr*1 gene expression is up regulated in *M. tuberculosis *engulfed by macrophages activated with IFNγ. On the other hand, Acr2 is the most up regulated protein under heat stress and induced in *M. tuberculosis *infecting quiescent macrophages [7]. Acr3 has not been examined in detail.

Based on amino acid homology, mycobacterial sHsps were classified into three groups [6]. *M. leprae *sHsp is a class 3 heat shock protein and homologs of class 3 sHsps are found in *M. smegmatis, M. marinum *and *M. avium *[8]. Surprisingly, *Streptomyces albus *encodes a single sHsp, which is 52% identical and 30% similar in amino acid sequence, making this the closest homolog to *M. leprae *sHsp18. This gene has been shown to be heat inducible in *S. albus *and confers marginal thermotolerence to the host [9].

*M. leprae *sHsp18 is represented among the antigenic targets of human T cell responses [10,11], and is presumably a secreted or a surface exposed antigen, since antibodies specific for this protein are found in the sera of lepromatous leprosy patients [12]. Our earlier report shows that the gene encoding sHsp18 is polymorphic and about 50% of the leprosy patients carry proline residue at the 52^nd ^position instead of serine [13]. However, there are no reports on the function of this protein either *in vitro *or *in vivo*. Earlier report predicting that sHsp18 could be a heat shock protein was based on indirect evidence. Monoclonal antibody (mAbL5) against *M. leprae *sHsp18 cross reacted with a protein of similar size only from *M. habana *among the other mycobacteria tested. This protein, presumably similar to sHsp18 of *M. leprae*, could be induced at 45°C [14]. Other stress factors such as ethanol, H_2_O_2 _and nalidixic acid did not induce this protein. In addition, the *in vivo *role of sHsp18 has not been demonstrated directly as of now. However, the promoter segment carrying 136 bp upstream region along with 29 codons of N-terminal region was activated in macrophages, when this fragment was cloned in a promoter probe vector and expressed in *M*. *bovis *BCG strain [15]. This indicates that sHsp18 is likely to be a stress inducible protein having a role in pathogenesis.

In this study, we have cloned and expressed the recombinant sHsp18 in *E. coli *in order to study the nature of the protein with respect to its function. The multimerisation and chaperone activity of the protein was also examined.

## Results

### Cloning and expression of *M. leprae *sHsp18 in *E. coli*

The gene encoding sHsp18 was cloned in the expression vector pQE31 to create pSA6, which encoded sHsp18 as N-terminal His-tag fusion protein. The protein was purified using nickel affinity column as described under experimental procedures. The purified protein migrated as a major band with a molecular weight of approximately 21 kDa (See Additional file. 1A). An additional band of molecular weight of 19 kDa was also observed. Western blot analysis (See Additional file. 1B) shows that the anti-sHsp18 antibodies recognized both proteins. The calculated molecular weight of the protein in pSA6 is 19.3 kDa including the amino acids added during construction. Exact molecular weights obtained using Mass Spectrometry showed a major protein of 19.3 kDa and a minor protein of 16.7 kDa. MALDI-TOF analysis of the tryptic digests of these two proteins excised from SDS-PAGE gels confirmed that both proteins were sHsp18 of *M. leprae *(Mascot score of 52 and 59 and sequence coverage of 36% and 38%, for the 19.3 kDa and 16.7 kDa proteins, respectively).

### Localization of sHsp18 in *E. coli*

**Figure 1:**
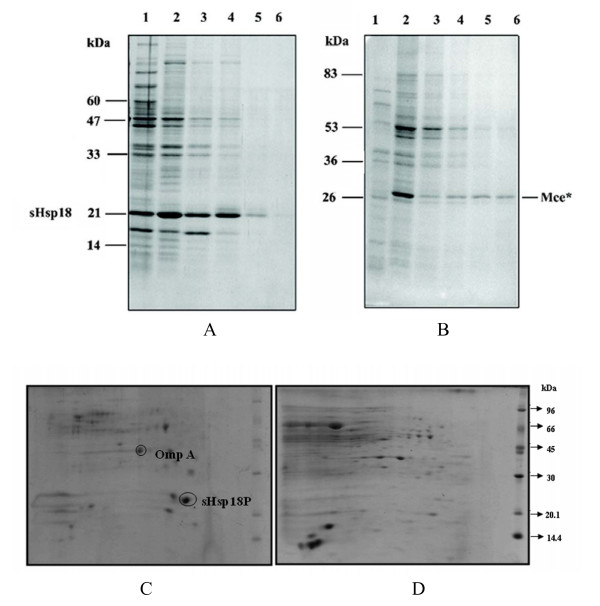
Comparison of the localization and solubilisation in urea of sHsp18 protein (A) and Mce* protein (B). To check whether the sHsp18 protein forms inclusion bodies on over expression, the protein profile of the soluble and insoluble protein fractions were checked on a 12% SDS-PAGE. Lanes contain the soluble fraction (lane 1), the insoluble fraction (lane 2) of the crude sonication extract, and the supernatants after incubation of the insoluble fraction with suspension buffer containing 2 M urea (lane 3), 4 M urea (lane 4), 6 M urea (lane 5), and 8 M urea (lane 6). C&D. 2D gel analysis to show the presence of sHsp18 in the outer membrane fractions of *E. coli*. 45 μg protein was loaded on a 7 cm IPG strip with a pI range covering 4–7. After the second dimension, the gel was stained with colloidal coomassie. sHsp18 and OmpA (marker for outer membrane fraction) have been shown. 'C' represents outer membrane fraction and 'D' represents inner membrane fraction.

During the extraction of sHsp18 from induced cultures, it was found that the protein was in 3000 rpm pellet indicating that the protein might be forming insoluble inclusion bodies. However, further analysis showed that sHsp18 could be extracted even in 2 M urea (Fig. 1A), unlike other inclusion bodies (Fig. 1B). Therefore, the localization of the sHsp18 was examined. Results clearly show that sHsp18 was found predominantly in the outer membrane fraction of *E. coli *and a small fraction was consistently found in the periplasm as well (Fig. 1C and Additional file 2.). However the protein was not detected in inner membrane and cytoplasmic fractions (Fig. 1D and Additional file 2). Absence OmpA in periplasmic fractions of 2D gels excludes the possibility of contaminating sHsp18 and the purity of the membrane fractions was evident by the absence of sHsp18 in inner membrane and cytoplasmic fractions (data not shown).

### sHsp18 forms oligomeric structures in solution

**Figure 2:**
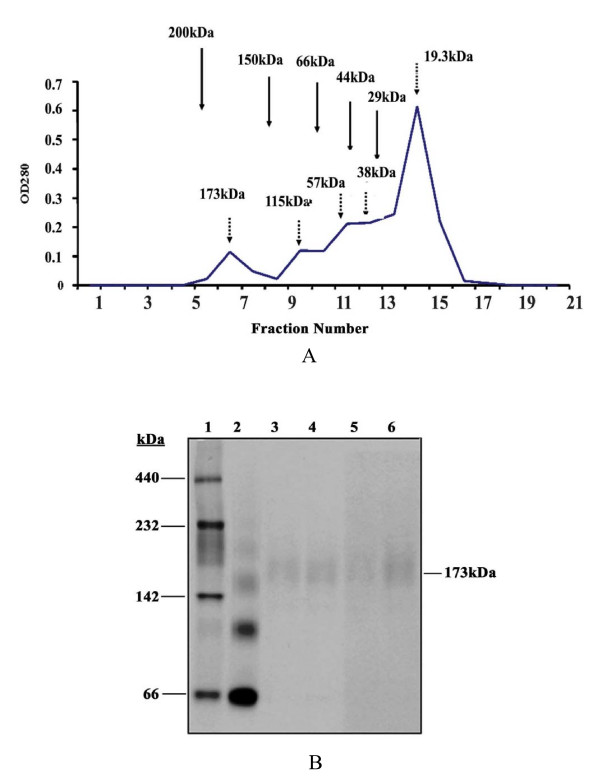
**(A). Self aggregation of sHsp18**. sHsp18 protein was purified by denaturing method and dialyzed against 50 mM PBS containing 200 mM NaCl for 12 hrs. Dialyzed protein was precipitated by acetone precipitation. The protein was dissolved in 1.5 M urea and applied to the column equilibrated against PBS and eluted the fractions in PBS. The bands corresponding to oligomeric forms were indicated by dotted arrows and position of molecular weight markers are indicated with solid arrows. (B). Non-denaturing Gel analysis of MagneHis Purified sHsp18 protein. Confirmation of the oligomeric form was done by native gel electrophoresis. Lanes represent High Molecular weight marker (lane 1), 10 μg of Non-denatured BSA showing oligomers (lane 2), 5 and 10 μg of purified protein under non-denatured condition (lanes 3–4), 5 and 10 μg of purified protein under denatured condition (lanes 5–6).

Small heat shock proteins form multimers of trimers or dimmers [16], and these oligomeric structures have been shown to be the functional forms [17]. In order to examine the status of sHsp18 in solution, the purified protein was dialyzed against PBS and NaCl for six hours and fractionated on sephacryl column. The protein was eluted in fractions corresponding to molecular weights ~173 kDa and ~115 kDa. However, extended dialysis against PBS led to the formation of a single peak at 173 kDa size indicating the formation of nonamers (data not shown). In order to examine the presence of intermediates of the oligomeric assembly, the dialyzed protein was adjusted to 1.5 M urea and then fractionated as described above. As shown in Fig. 2A, various intermediate forms of dimer, trimer hexamers and nonamers could be detected. SDS-PAGE analysis of the individual fractions showed the presence sHsp18 protein and the assembly process were further confirmed by gluteraldehyde cross linking (data not shown). The size of this nonameric forms were confirmed against standard molecular weight markers on a native gel (Fig. 2B). The protein might have post-translational modifications and hence the resolution of native gels is not that good, only a diffuse band could be seen. However, repeated experiments confirm the results shown in Fig. 2B.

### sHsp18 can act as molecular chaperone *in vitro*

**Figure 3:**
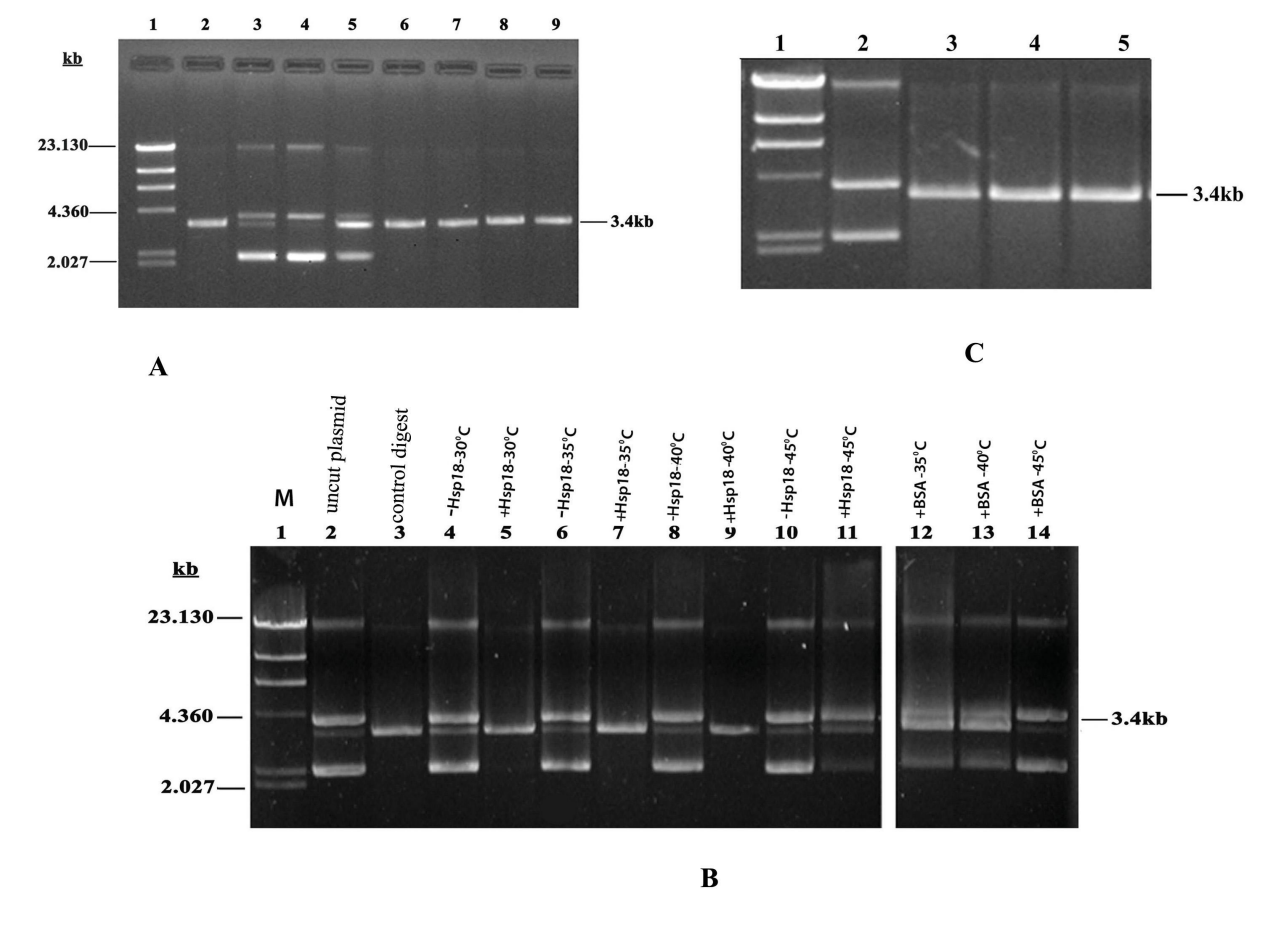
**(A). Comparison of *in vitro* chaperone activity of α-crystallin and purified sHsp18**. Enzymes (2 U) were heat inactivated (*Sma*I at 37°C for 90 min and *Nde*I at 45°C for 90 min) in the presence or absence of molecular chaperones (0.2 μg) and assayed for the cleavage of 1 μg of plasmid DNA. Lanes represent, λ*Hind* III marker (lane 1), digested plasmid (lane 2), uncut plasmid (lane 3), plasmid digested with- heat inactivated *Sma*I (lane 4), heat inactivated *Nde*I (lane 5), heat inactivated *Sma*I in the presence of sHsp18 (lane 6), heat inactivated *Sma*I in the presence of α-crystallin (lane 7), heat inactivated *Nde*I in the presence of sHsp18 (lane 8) and heat inactivated *Nde*I in the presence of α-crystallin (lane 9). **(B). sHsp18 can act as a molecular chaperone in wide range of physiological temperatures.** 0.2 μg sHsp18 or BSA was incubated with 2 U of *Sma*I at different temperatures for 90 min, and the cleavage of plasmid DNA was assayed at 25°C for 3 hrs. Lanes represent, λ*Hind* III marker (lane 1), uncut plasmid (lane 2), plasmid digested with *Sma*I as control (lane 3), plasmid incubated with heat inactivated *Sma*I without and with sHsp18 at 30°C (lanes 4–5); at 35°C (lanes 6–7), at 40°C (lanes 8–9), at 45°C (lanes 11–12), plasmid incubated with *Sma*I with BSA at 35°C, 40°C and 45°C (lanes 12–14). **(C). Preheating of molecular chaperones does not affect chaperone activity.** Lanes represent, λ*Hind* III marker (lane 1), undigested plasmid (lane 2), plasmid digested with- *Sma*I (lane 3), *Sma*I with preheated sHsp18 at 100°C for 5 min (lane 4) and with preheated α-crystallin at 100°C for 5 min (lane 5).

Prevention of thermal inactivation of restriction enzymes was used earlier to demonstrate the chaperone function of α-crystallin [18]. This assay was validated first by measuring the chaperone activity of α-crystallin under our experimental conditions. As shown in Fig. 3A (tracks 7 and 9), α-crystallin prevents the thermal inactivation of *Sma*I and *Nde*I. Tracks 6 and 8 show that co-incubation of sHsp18 and the restriction enzymes prevented the thermal inactivation, implying that sHsp18 protein is as good as α-crystallin in its chaperone function. The molar ratio of the substrate to chaperone could not be calculated since the exact protein concentration of the commercial enzyme could not be determined.

Further, we have analyzed the temperature range at which sHsp18 could prevent thermal denaturation. From Fig. 3B, it is clearly seen that sHsp18 at a concentration of 0.2 μg could prevent the thermal aggregation of *Sma*I at temperatures ranging from 30°C to 40°C. However, at 45°C, it is ineffective in preventing the inactivation, whereas, 0.2 μg of BSA could not prevent the thermal inactivation of *Sma*I.

For checking whether the denatured sHsp18 has chaperone activity, the protein was incubated in boiling water bath for 5 min before adding to the assay mixture. From Fig. 3C, it is clear that preheating does not affect the chaperone function of sHsp18.

### Physical Interaction of sHsp18 with *Sma*I protein

**Figure 4:**
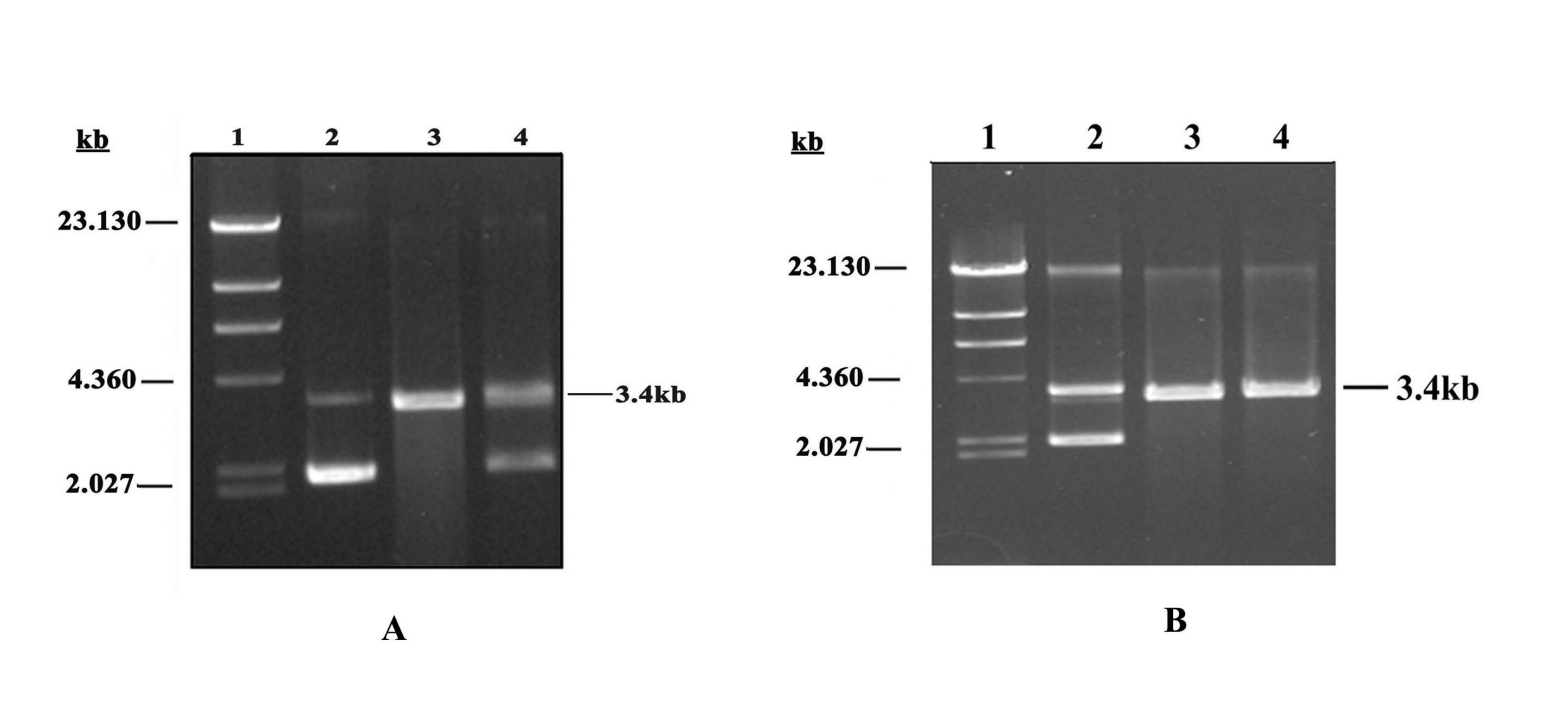
**(A). sHsp18 interacts with *Sma*I**. Heat inactivated *Sma*I was added to, sHsp18 bound MagneHis particles as per methods. sHsp18 was able to interact and refold the heat inactivated *Sma*I and restore its biological activity. But in the control, heat inactivated *Sma*I without sHsp18 did not show any activity. Lanes represent, λ Hind III marker (lane 1), undigested plasmid (lane 2), plasmid DNA digested with heat inactivated *Sma*I added to sHsp18 bound MagneHis particles (lane 3), plasmid DNA treated with heat inactivated *Sma*I added to MagneHis particles (lane 4). (B) *Sma*I do not bind directly to MagneHis particles. Native *Sma*I was incubated with MagneHis particles for 5 min and the supernatant and pellet fractions were taken for restriction assay. Lanes represent, λ *Hind *III marker (lane 1). Plasmid DNA was digested with *Sma*I added MagneHis pellet fraction (lane 2), plasmid DNA digested with supernatant fraction (lane 3) and control digest (lane 4).

Histidine tagged sHsp18 was bound to MagneHis particles as described in experimental procedures. One dimensional gel analysis confirmed the presence of only sHsp18 protein bound with MagneHis particles. After removal of non specific proteins, heat inactivated *Sma*I was added to the sHsp18 bound MagneHis particles and incubated at 37°C for 30 min. The mix was washed extensively to remove nonspecifically bound *Sma*I. After washing, substrate plasmid DNA was added to the magnetic beads containing bound sHsp18-*Sma*I complex. As shown in Fig. 4A, the plasmid DNA was cleaved, indicating that *Sma*I was retained on the beads presumably through its interaction with sHsp and also regained its biological activity due to the chaperone function of sHsp18. In the control experiment, incubation of heat denatured *Sma*I with MagneHis particles, without bound sHsp18, did not result in binding of the restriction enzyme. Similarly, native *Sma*I under the same experimental conditions failed to bind to the MagneHis particles. These control experiments rule out the possibility of nonspecific binding of *Sma*I to MagneHis particles (Fig. 4B). However, *Sma*I, heat inactivated at and above 45°C, was unable to bind to sHsp18 (data not shown), which implies that completely denatured proteins could not be refolded to active enzyme by sHsp18.

## Discussion

### Localization of sHsp18 in *E. coli*

Small heat shock proteins are a family of stress proteins, which were shown to act as molecular chaperones *in vitro *and they also play a critical role in disease conditions [1,19]. sHsps are often up regulated in neurodegenerative diseases, and also in motor neuron cell injury [1,20]. The sHsp18 of *M. leprae *is a major T cell antigen [11,21], which comes under small heat shock protein family. It has been shown that sHsp18 could be used as a diagnostic marker for *M. leprae *infection [22-24]. Although *M. leprae *genome has undergone reductive evolution, it still retains the sHsp18 gene [25]. Our observation showed that sHsp18 gene is expressed even in tuberculoid end of the spectrum [13]. This prompted us to examine the function of the protein encoded by this gene. Since *M. leprae *is an uncultivable organism, we have cloned and expressed this protein as a fusion protein in *E. coli*. Western blot analysis of gel separated proteins and MALDI-TOF analysis shows the presence of two major forms of this protein. In addition, MALDI-TOF analysis of the purified sHsp18 in the linear mode shows two peaks for the 19.3 kDa form of the protein (data not shown). Further experiments are needed to determine the reason for this. Many proteins, which are over expressed in *E. coli*, are shown to generally form inclusion bodies [26]. However, our studies show that sHsp18 is expressed as a soluble protein. Localization of the protein in *E. coli *showed that the major proportion of the protein was seen in membrane fraction and some amount in periplasmic fractions, but not in cytoplasmic and inner membrane fraction. Further, since the survival of the recombinant *E. coli *over expressing the sHsp18 is not altered (unpublished results), cell lysis is unlikely. These results clearly show that sHsp18 is localized in the outer membrane of *E. coli*. However, *E. coli *being a gram-negative organism, this data needs confirmation using another mycobacterial cloning host. Earlier reports [27] showed the presence of sHsp18 in cell wall and membrane fraction of armadillo derived *M. leprae*. Membrane associated sHsp18 could be important for modulating the immune response to infection since membrane bound but surface exposed epitopes could induce T cell as well as B cell responses (19), apart from their possible involvement in membrane stabilization as has been shown for the sHsp17 of *Synechocystis *[29]. In fact, the sHsp 17 of *Synechocystis *was shown to be an amphitrophic protein. However, since the sHsps do not have signal sequences or transmembrane region, the mechanism of their transport is unclear. Mambula *et al*. suggested three possible mechanism of release of Hsp60 by non canonical secretion [30].

### Self aggregation of sHsp18

Oligomerisation of small heat shock proteins has been reported to be essential for protein stability, which in turn enhances chaperone activity and protein-protein interactions. Small heat shock proteins usually exist as oligomers of variable sizes, ranging from 150 kDa to 800 kDa, with each subunit being 12–42 kDa, and appear to undergo dynamic dissociation/reassociation, with oligomeric dissociation being a prerequisite for their chaperone activities [31]. sHsp16.3 of *M. tuberculosis *is reported to form nonamers, which are assembled and reassembled via trimer and hexamer intermediates [16,32]. Native form of this protein was not found to be essential for chaperone activity of the protein [33]. Our results show the formation of oligomeric forms of sHsp18 by gel filtration analysis. These forms correspond to the molecular weight of monomer, dimer, trimer, hexamer and nonamers. Complete removal of urea by dialysis showed only a single peak of nonamers. But partial removal shows the hexameric form also. Presence of trimeric forms indicates that the monomeric forms can probably form trimeric intermediates, which lead to the formation of nonameric forms through hexamer intermediates. We could see dimeric intermediates also in the assembly process, whose significance in the assembly process is yet to be clarified. However, further studies are needed to establish the structural organization of these oligomers and the interactions between specific domains that characterize these assemblies. And, also it would be interesting to examine the state of the protein *in vivo*.

### sHsp18 functions as a molecular chaperone *in vitro*

Small heat shock proteins have been reported to prevent the thermal aggregation of restriction enzymes, such as *Nde*I and *Sma*I, and help in retaining their biological activity [18]. In this experiment, we have analyzed the role of sHsp18 in preventing the heat denaturation of *Sma*I and *Nde*I. Following heat treatment, the enzymatic activity was assayed using plasmid DNA as substrate. Thermal inactivation experiments showed that sHsp18 could prevent the enzymes from heat inactivation indicating their ability to act as chaperone. Interestingly, sHsp18 acts as a chaperone at a concentration much lower than α-crystallin. Further, sHsp18 could also enhance the biological activity of these enzymes (data not shown).

Molecular chaperone activity was not lost even after the pre treatment of the sHsp18 at 100°C for 5 min. Similar finding was reported for *M. tuberculosis *Hsp16.3 as well [34]. Protein stability is one of the most important obstacles for successful formulation in the development of new generation vaccines [35,36]. sHsp18 of *M. leprae *was shown to be used as a successful vaccine delivery system [37]. However, the prevention of thermal inactivation of *Sma*I occurs only up to 40°C. This may be due to the fact that complete denaturation of the *Sma*I may be occurring at higher temperature [9] and sHsp18 may be able to act only on partially denatured proteins.

### Interaction of sHsp18 with *Sma*I

Assembly into oligomeric structures apparently is essential for the activity of sHsps *in vivo*, since the protein oligomerises *in vitro*. Known chaperones do not possess steric information for protein folding but inhibit unproductive folding and assembly pathways which would otherwise act as dead-end kinetic traps and produce incorrect structures. Protein-protein interaction is an important aspect of the chaperone function. In our studies we were able to show that sHsp18 could physically interact with *Sma*I and was able to restore the biological activity.

## Conclusion

Data in this report show that the small heat shock protein sHsp18 of *M. leprae *is a molecular chaperone and shows several properties associated with other small heat shock proteins. sHsp18 can be a useful protein as it has remarkable ability to refold proteins which have partially lost their biological activity. This protein is also shown to be capable of forming high molecular weight aggregates and taking part in protein-protein interactions. Localization of the protein to the membrane compartment (when over expressed in *E. coli*) is another significant discovery. These properties may help the pathogen carrying this protein in its survival under different stress conditions that they may possibly encounter after infection. Direct role of molecular chaperons in infection and immunity is well documented [28,38]. Membrane association of sHsp18P suggests a possible role of this protein in protecting the cell and also in modulating the host immune response. Since *M. leprae *has undergone reductive evolution and thereby lost most of its functional genes, the retention of sHsp18 implies the importance of this protein in the survival and perhaps virulence of the bacteria.

## Methods

### Cloning and expression of the *shsp18 *gene of *M. leprae *in *E. coli*

Punch biopsies from lepromatous leprosy patients were collected after obtaining the informed consent as per the institutional ethical committee and Indian council of medical research. Full length *shsp *gene [Acc. No. ML 1795] of *M. leprae *was amplified from biopsy-derived cDNA of leprosy patients as described earlier [13]. The amplified fragment was cloned into pGEM-T vector (Invitrogen) and the recombinant plasmid (pSA6) isolated from *E. coli *transformants was digested with *Sph*I and *Pst*I, and the released fragment was sub cloned in to pQE31 expression vector (Qiagen). Recombinant clones were checked for size increase and confirmed by sequencing. For induction experiments, the culture was grown in 3 mL LB containing 100 μg/mL ampicillin and 25 μg/mL kanamycin, at 37°C overnight. Expression of the recombinant protein was induced with isopropyl thio-β-D-galactoside (IPTG) added to a final concentration of 0.4 mM when the culture reached an A_600 _of 0.6. After four hours of growth, the induced cells were harvested by centrifugation, washed once with PBS and the pellet used for further experiments.

### Purification of protein using Ni-NTA resin and MagneHis particles

The His-tag containing protein was purified using Ni-NTA affinity chromatography under native or denaturing conditions. Pellets were resuspended in lysis buffer (8 M urea, 0.1 M Na-phosphate, 0.01 M Tris-Cl, pH 8.0) and the purification was done according to manufacturer's instruction using Ni-NTA resin (Sigma).

Purification using MagneHis particles (Promega) was done as per the manufacture's instruction. Briefly, 1 mL of the induced culture was spun down and washed once with PBS. The cell pellet was resuspended in 150 μl of 1× FastBreak Cell Lysis Reagent (Promega) and 1 μl DNase I was added and the suspension was incubated for 20 min at room temperature on a rotary mixer. Binding buffer150 μl (100 mM HEPES, 10 mM imidazole, 500 mM NaCl) was added to the cell lysate followed by 50 μl of MagneHis Ni-Particles (Promega), and incubated for 5 min at room temperature. The tubes were exposed to magnetic field for 30 seconds to capture the MagneHis Ni-Particles. The supernatant was carefully removed and 200 μl of Magne-Binding buffer was added and mixed. The tubes were exposed to magnetic field as before and the supernatant was removed and discarded. The wash step was repeated two more times. Bound proteins were eluted using 100 μl of Elution Buffer (100 mM HEPES, 500 mM imidazole, pH 7.5), as described by the supplier. Binding and elution buffer contained 8 M urea for the purification of protein under denaturing condition. Protein concentration was estimated using Bradford's method.

### Immunodetection

Polyclonal rabbit anti-serum was raised against purified recombinant *M. leprae *sHsp18 protein (500 μg/injection) by subcutaneous immunization of rabbits. Blood (20 mL) was collected and left undisturbed at room temperature for 1 hr. This was further incubated at 4°C overnight and centrifuged at 2500 rpm for 30 min at 4°C. Upper aqueous layer was collected and further clarified at 2500 rpm for 30 min at 4°C. An equal volume of ammonium sulphate was added to the supernatant and, adjusted to pH 7.2 with ammonium hydroxide. The mixture was kept at 4°C for 6 hrs. The suspension was spun at 3000 rpm for 30 min and the supernatant was stored at -20°C. Protein samples electrophoresed in 12% SDS-polyacrylamide gels were either stained with coomassie blue or transferred to nitrocellulose membrane. The membrane was blocked for 2 hrs in 40 mL TBS-T blocking buffer containing 5% (w/v) milk powder, followed by overnight incubation at 4°C with anti-sHsp18 antibody (1:1000 dilution in TBS buffer). Blots were washed thrice in TBS-T buffer and once in TBS, prior to incubation for 45 min with goat anti-rabbit IgG-peroxidase conjugate (Genei, Bangalore) diluted in TBS buffer containing 0.1% milk powder. Membranes were washed as above and used for staining with DAB solution.

### MALDI-TOF mass spectrometry

Stained gel bands from SDS-PAGE were cut into one-mm^2 ^small bits and destained by repeated washes in 25 mM ammonium bicarbonate. The gel pieces were dehydrated with acetonitrile and subjected to reduction using 10 mM DTT in 25 mM ammonium bicarbonate followed by alkylation in 55 mM Iodoacetamide in 25 mM ammonium bicarbonate. After three washes with 100 mM ammonium bicarbonate, the gel pieces were dehydrated using acetonitrile, dried and rehydrated with 5 μl of trypsin (400 ng) in 100 mM ammonium bicarbonate in 10% ACN. The gel pieces were incubated at 37°C for 16 hrs. After overnight digestion, the supernatant was collected, 25 μl of 0.1% TFA in 60% ACN was added, sonicated in a bath sonicator for 3 min and incubated at room temperature for 10 min. The supernatant was collected and pooled to the previous one. The gel pieces were dehydrated with ACN, vortexed and incubated at room temperature for 10 min. The supernatant was collected and pooled. The extracted peptides were then completely dried in a speed vac. The dried peptides were resupended in 5 μl of 0.1%TFA in 5% ACN and purified using C-18 micro columns (Eppendorf) according to the manufacturer's instructions. The desalted peptides were eluted in 5 μl of 0.1% TFA in 50% ACN. The purified peptides were then taken for spotting. α-Cyano-4-Hydroxy Cinnamic acid (CHCA) matrix (10 mg/mL) was made in 70% acetonitrile and 0.03% TFA. Another matrix used was 2 mg/mL of CHCA with 50 mM ammonium monobasic phosphate as additive. Peptide samples were applied on a stainless steel MALDI target plate using the sandwich method. When CHCA matrix was used, 0.5 μl of matrix was first applied on the plate, allowed to dry. Sample (0.5 μl) was applied and then immediately layered on top with 0.5 μl of matrix. Sample was diluted 5 fold in CHCA matrix containing ammonium phosphate, and 1 μl of the sample was applied. Peptide mass spectrum was acquired using Axima CFR plus (KRATOS Shimadzu) MALDI-TOF mass spectrometer in the reflectron mode. The acceleration voltage after pulsed extraction is 20,000. The instrument was calibrated using external standards Bradykinin (757.39 Da), Angiotensin II (1046.54 Da), P14R (1533.85 Da) and ACTH fragment 18–39 (2465.19 Da). Peak list was generated without using the smoothing function. The signal to noise ratio of 20 and above was used for database search. The monoisotopic masses were processed for identification by using the search engines, MASCOT . Search was performed in NCBInr, MSDB and Swiss-Prot. The search parameters were as follows: mass tolerance – 0.05 to 0.5 Da, Species – *E. coli*, *Mycobacterium*, and maximum missed cleavages was set to 1, fixed modification – carbamidomethyl (for cysteine modification by Iodoacetamide), variable modifications – oxidation of Methionine and Propionamide (for cysteine modification by acrylamide). The molecular masses of the purified sHsp18 protein were determined by MALDI-TOF mass spectrometer operating in linear mode. For this purpose, the pure protein in water (5 pmoles) was mixed with equal volume of Sinnapinic acid (10 mg/mL in 33%ACN and 0.067% TFA) and 1 μl was spotted, dried and analyzed.

### Localization of sHsp18 protein in *E. coli*

To check whether sHsp18 forms inclusion bodies, 10 mL culture cell pellet was resuspended in 1 mL of sonication buffer (300 mM NaCl, 50 mM Tris-Cl, pH 8.0), lysed by sonication (Vibracell, USA), and then subjected to centrifugation at 12000 rpm for 7 min at 4°C. The supernatant was collected and the pellet extracted sequentially using different concentrations of urea. The supernatant and pellet fractions after each urea extraction step were checked for the presence of the sHsp18 protein on a 12% SDS-PAGE. As a control, similar experiment was done using a recombinant clone encoding the Mce* protein of *M. leprae*. Mce* is the N-terminal truncated form (Mw = 26 kDa) of the Mammalian Cell Entry (Mce) protein of *M. leprae*. The mce* gene was cloned in the pQE31 vector and has been previously shown to form inclusion bodies upon over expression by IPTG induction [39].

Sub cellular fractionation was performed as described earlier [40] with the following modifications. Cell pellets from induced cultures (20 mL) were washed twice in PBS and resuspended in equal volume of spheroplast buffer [50 mM Tris (pH 8.0), 18% sucrose, 1 mM CaCl_2_, 0.5 mM EDTA and 0.5 μg/mL lysozyme]. After 20 min incubation, the cells were spun at 10000 rpm for 15 min. The supernatant (periplasmic fraction) was stored at -20°C. The pellet was resuspended in 2 mL of TE [50 mM Tris (pH8.0), 2.5 mM EDTA] buffer and sonicated. The lysate was centrifuged at 10000 rpm for 4 min at 4°C. The pellet, mainly containing unbroken cells, was resuspended in 200 μl of SDS lysis buffer. The supernatant was centrifuged at 150,000 × g for 40 min at 4°C and the supernatant (cytoplasmic fraction) was stored at -20°C. The pellet was resuspended in 1 mL of TE buffer with 2% sarkosyl (N-lauryl-sarkosine). Incubated at 25°C for 20 min and centrifuged at 50000 rpm for 40 min at 4°C. The supernatant (inner membrane fraction) was collected and stored at -20°C and the pellet was resuspended in 200 μl SDS lysis buffer (outer membrane fraction). The fractions were separated and analyzed using 1D and 2D gel electrophoresis.

### 2D gel analysis

Protein concentration was estimated using Bradford's method and analyzed in 2D gel as per earlier reported protocol with slight modifications [41]. Samples were solubilised in 7 M urea, 2 M thiourea 4% CHAPS, 1% ampholytes, 100 mM DTT, and 0.004% bromophenol blue, for subsequent IEF. Commercial IPG strips (GE healthcare) were rehydrated at 20°C overnight with sample solutions using an IPGphor IEF apparatus (GE Healthcare) according to manufacturer's instruction. IPG strips were equilibrated with equilibration buffer (6 M urea, 2 M thiourea, 50 mM Tris, 30% glycerol, 2% SDS, 0.005% BPB, 1%DTT for 15 min followed by 4.8% iodoacetamide replacing DTT in the second step) and analyzed in 7 cm, 12.5% SDS-PAGE using Mini Protean Dodeca cell apparatus (Biorad). Gels were stained with colloidal coomassie, analyzed by Image master 2D platinum version 5.0 software (GE Healthcare). Spots were excised from for In-Gel Tryptic digestion and analyzed by MALDI-TOF spectrometry [41].

### Self-aggregation of sHsp18

Size exclusion chromatography of purified sHsp18 was performed on a Sephacryl H 200 16 × 20 column (BioRad). The recombinant sHsp18 protein was purified by Nickel affinity chromatography using denaturing method. Purified protein was dialyzed against 50 mM PBS containing 200 mM NaCl, pH 7.2 at 4°C for 6 hrs. The dialyzed protein was precipitated by acetone and resuspended in PBS and applied to the column equilibrated with the same buffer. For separating the different intermediate forms of oligomers, precipitated protein was dissolved in 1.5 M urea and applied to the column. Fractions of 1 mL were collected at a flow rate of 0.5 mL/min. The column was calibrated with high molecular weight standards-α-Amylase (200 kDa), Alcohol Dehydrogenase (150 kDa), Bovine Serum Albumin (66 kDa), Ovalbumin (44 kDa) and Carbonic Anhydrase (29 kDa) (Sigma Aldrich.). Precipitated proteins from various fractions were examined using SDS-PAGE.

### Native gel electrophoresis

Recombinant sHsp18 protein purified by MagneHis Protein purification system was analyzed in a non-denaturing polyacrylamide gel (7.5%). High molecular weight calibration kit for native electrophoresis (GE Health care) were used – thyroglobulin (669 kDa), ferritin (440 kDa), catalase (232 kDa), lactate dehydrogenase (140 kDa), and BSA (66 kDa). Gels were stained with Coomassie Blue.

### Determination of chaperone activity

Chaperone activity was assayed as reported earlier [18]. In this assay, restriction enzymes *Sma*I and *Nde*I (New England Biolabs, Beverly, MA) were used, according to the manufacturer's recommendations. Two units of enzyme was used to digest 1 μg of plasmid DNA (pQE31). For heat inactivation, *Sma*I was incubated at 37°C for 90 min and *Nde*I at 45°C for 90 min with or without sHsp18 or α-crystallin.

### Analysis of physical interaction of sHsp18 and *Sma*I

One mL of induced culture of *E. coli *culture was pelleted, lysed using 150 μl of 1× Fast Break cell lyses solution (Promega) as described earlier. To this lysed solution, 150 μl of binding buffer was added and the lysate was mixed with 30 μl MagneHis particles (Promega) and incubated at room temperature for 5 min. Wash buffer (150 μl, 100 mM HEPES, 10 mM Imidazole, 500 mM NaCl, pH.7.5) was added to remove unbound proteins and the washing was repeated for three times. Heat inactivated *Sma*I (five units) was added and incubated at 37°C for 30 min. The MagneHis particles were washed extensively to remove all nonspecific proteins and then the substrate plasmid DNA was added. Restriction digestion was carried out at 25°C for 3 hrs. For checking the nonspecific binding of *Sma*I to the MagneHis particles, native *Sma*I was added to MagneHis particles without sHsp18, under the same experimental conditions, and incubated at room temperature for five minutes. Supernatant fraction and pellet fraction having MagneHis were collected and restriction digestion was carried out in both the fractions separately.

## Authors' contributions

NL contributes to oligomerisation of sHsp18, chaperone studies and protein interaction studies, EAR and SS carried out the localization studies, JJ performed the inclusion body analysis of sHsp18 and NPS participated in the clinical aspects including collection of biopsy samples and in the study design. KD conceived of the study, and participated in its design and coordination. All authors read and approved the final manuscript.

## Supplementary Material

Additional file 1 **Purification and identification of sHsp18 of *M. leprae *in *E. coli***. (A) SDS-PAGE showing the expression of sHsp18 in *E. coli*. The protein profile of the *E. coli *cells over expressing the sHsp18 protein were checked on a 12% SDS-PAGE before and after IPTG induction. Lanes represent, Molecular weight marker (lane 1), Total cell lysate, uninduced and induced (lanes 2–3), Purified sHsp18 (lane 4). (B) Western blot analysis was done to confirm the identity of the protein Click here for file

Additional file 2 **Localization of sHsp18 in *E. coli***. To determine the localization of the sHsp18 protein on expression in *E. coli*, the cells were fractionated and the protein profile of each fraction was checked on a 12% SDS-PAGE for the presence of the sHsp18 protein. Lanes represent 1-Purified sHsp18 protein (Control), 2-Periplasmic fraction, 3-Cytoplasmic fraction, 4-Inner membrane fraction, 5-Outer membrane fraction. Positions of molecular weight markers were shown on the side of the gel. Click here for file
