# Supplementary figures and images for: Functional characterization of a small heat shock protein from Mycobacterium leprae
Source: BMC Microbiol. 2008 Nov 28;8:208. doi: 10.1186/1471-2180-8-208 (PMC2629775; doi:10.1186/1471-2180-8-208)

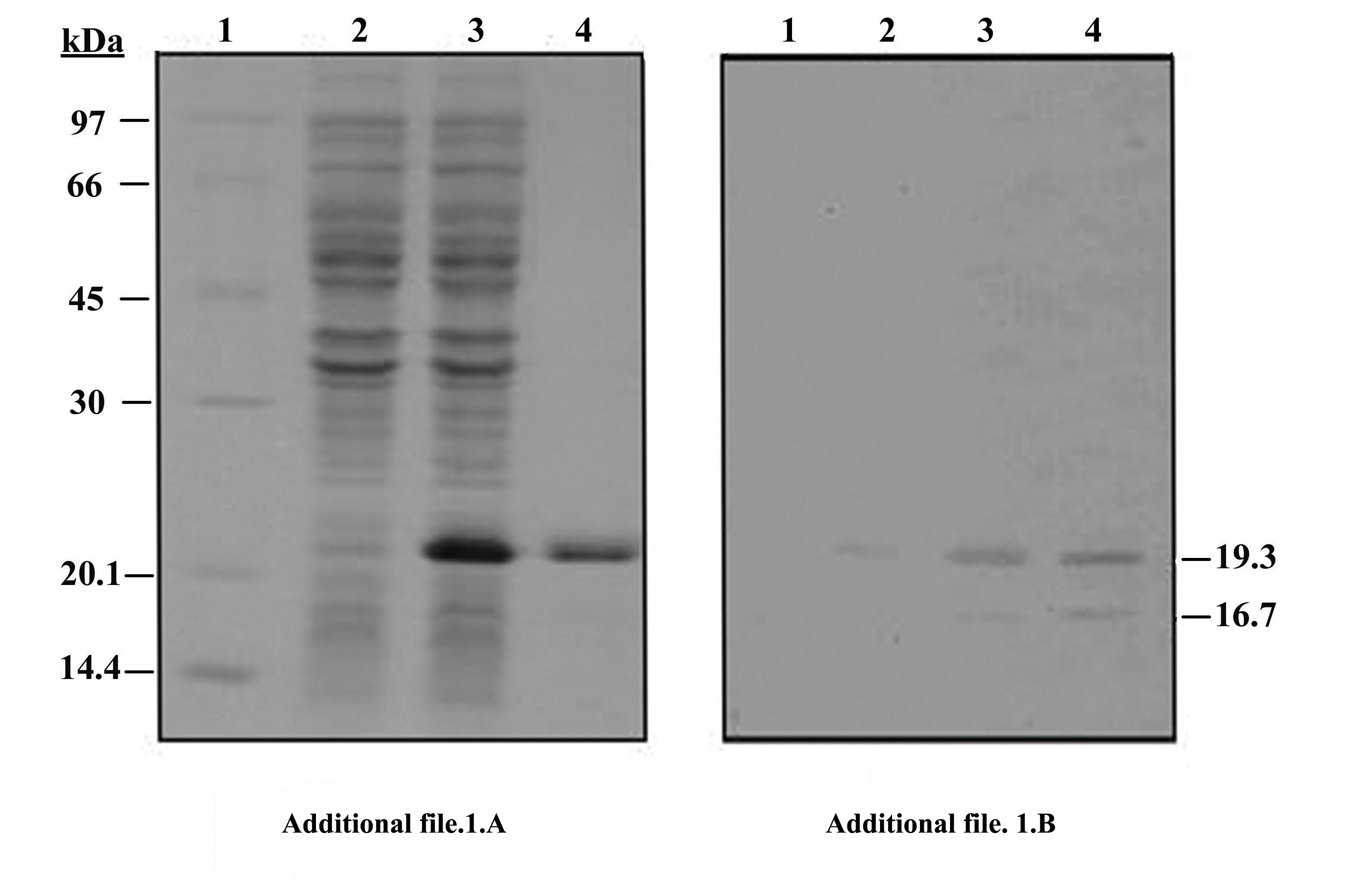

Supplement: Additional file 1 — Purification and identification of sHsp18 of M. leprae in E. coli. (A) SDS-PAGE showing the expression of sHsp18 in E. coli. The protein profile of the E. coli cells over expressing the sHsp18 protein were checked on a 12% SDS-PAGE before and after IPTG induction. Lanes represent, Molecular weight marker (lane 1), Total cell lysate, uninduced and induced (lanes 2–3), Purified sHsp18 (lane 4). (B) Western blot analysis was done to confirm the identity of the protein [file 1471-2180-8-208-S1.jpeg]

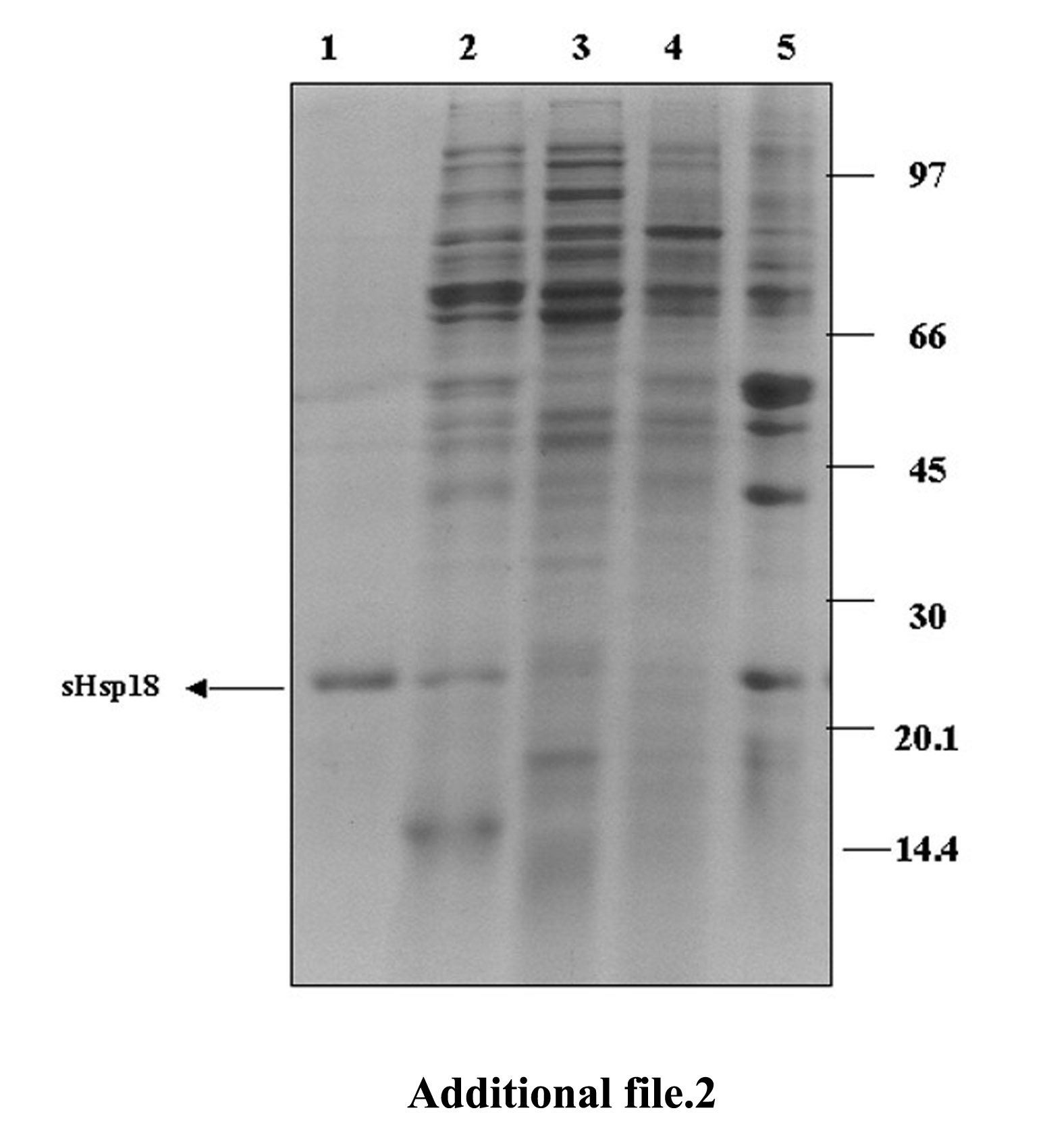

Supplement: Additional file 2 — Localization of sHsp18 in E. coli. To determine the localization of the sHsp18 protein on expression in E. coli, the cells were fractionated and the protein profile of each fraction was checked on a 12% SDS-PAGE for the presence of the sHsp18 protein. Lanes represent 1-Purified sHsp18 protein (Control), 2-Periplasmic fraction, 3-Cytoplasmic fraction, 4-Inner membrane fraction, 5-Outer membrane fraction. Positions of molecular weight markers were shown on the side of the gel. [file 1471-2180-8-208-S2.jpeg]
